# Sacha Inchi (*Plukenetia volubilis* L.) Oil Press-Cake Powder: Chemical Characterization and In Vitro Bioactivity for Sustainable Applications

**DOI:** 10.3390/molecules31010117

**Published:** 2025-12-29

**Authors:** Valeria Guarrasi, Barbara Prandi, Tullia Tedeschi, Benedetta Chiancone, Andrea Di Fazio, Raffaella Barbieri, Debora Baroni, Marilú Roxana Soto-Vásquez, Silvia Vilasi, Francesca Falco, Martina Cirlini, Daniel Paredes-López

**Affiliations:** 1 Institute of Biophysics, National Research Council (CNR), Via Ugo La Malfa 153, 90146 Palermo, Italy; silvia.vilasi@cnr.it; 2 Department of Food and Drug, University of Parma, Viale Parco Area delle Scienze 27/A, 43124 Parma, Italy; barbara.prandi@unipr.it (B.P.); tullia.tedeschi@unipr.it (T.T.); benedetta.chiancone@unipr.it (B.C.); martina.cirlini@unipr.it (M.C.); 3 Institute of Biophysics, National Research Council (CNR), Via De Marini 6, 16149 Genova, Italy; raffaella.barbieri@ibf.cnr.it (R.B.); debora.baroni@ibf.cnr.it (D.B.); 4 Facultad de Farmacia y Bioquímica, Universidad Nacional de Trujillo, Av. Juan Pablo II, Trujillo 13011, Peru; msoto@unitru.edu.pe; 5 Institute for Marine Biological Resources and Biotechnology (IRBIM)—CNR, L. Vaccara, 91026 Mazara del Vallo, Italy; francesca.falco@cnr.it; 6 Department of Animal Science, Universidad Nacional Agraria de la Selva, Carretera Central Km 1.21, Tingo María 10131, Peru; daniel.paredes@unas.edu.pe

**Keywords:** antinutritional factors, antioxidant activity, essential amino acids, fatty acids, Sacha inchi oil press-cake, total phenolic content, pharmacological potential

## Abstract

Sacha inchi (*P. volubilis* L.), an ancient oilseed crop native to the Amazon, is gaining attention for its high nutritional value particularly due to its ω-3-, -6-, -9-rich oil. However, most research has focused mainly on oil characterization, neglecting the potential of its by-products, such as the Sacha inchi oil-press cake (i.e., the solid residue after oil extraction). This study explores the chemical composition of Sacha inchi oil press-cake powder, focusing on fatty acid and amino acid profiles, antinutrient factors, total phenolic content, antioxidant activity, and the bioactivity of its extracts on cellular models. Fatty acid analysis revealed a high proportion of polyunsaturated fatty acids, especially α-linolenic acid (42.52%), making it a valuable resource for health-promoting applications. The protein content was also significant (41.86%), with a balanced amino acid composition, including essential amino acids such as leucine, valine, and isoleucine, which are vital for muscle protein synthesis and energy metabolism, in food and/or feed applications. Antinutritional factors were detected, including saponins (1050.1 ± 1.1 mg/100 g), alkaloids (2.1 ± 0.5 mg/100 g), and tannins (6.2 ± 0.9 mg/100 g). While these phytotoxins could limit their use in food applications, their potential antimicrobial activity highlights promising pharmacological opportunities. Total phenolic content (TPC) and antioxidant activity (AO) were evaluated using two extract mixtures differing in composition and polarity, with the acetone/water/acetic acid solvent (80/19/1 *v*/*v*/*v*) showing the highest antioxidant properties. The extract obtained showed cytotoxic effects against Panc-1 cancer cells, highlighting its potential in nutraceutical and pharmaceutical applications. This study underscores the unexploited potential of Sacha inchi by-products, such as the oil press-cake, as a sustainable resource of bioactive compounds for functional products, supporting circular bio-economy strategies by plant-based waste and local biodiversity valorization.

## 1. Introduction

Sacha inchi (*Plukenetia volubilis* L.), commonly referred to as the Inca peanut, is an ancient oilseed crop with a rich cultural history and promising industrial potential. Native to the Amazonian regions of Peru, it has been cultivated for over 3000 years, with archeological evidence linking its use to pre-Incan civilizations [1,2,3]. Traditionally consumed by indigenous communities in both raw and roasted forms, Sacha Inchi has gained increasing attention in recent decades due to its exceptional nutritional profile, particularly its high content of polyunsaturated fatty acids and proteins [4,5].

The Sacha Inchi (SI) seeds are especially valued for their oil, which contains α-linolenic acid (ALA, ω-3) and a favorable ratio of ω-3 to ω-6 fatty acids [5,6]. This composition not only makes it attractive for food applications, but also for use in cosmetics and pharmaceuticals, due to its anti-inflammatory and antioxidant properties [7]. Although Sacha Inchi oil has traditionally been employed for medicinal purposes and skincare by indigenous peoples [7,8,9], its commercial production has increased significantly in the 21st century, with cultivation spreading across South America and Southeast Asia [10,11]. Despite its well-documented health benefits and growing commercial interest, scientific research has focused primarily on the oil.

SI by-products, such as raw seeds, leaves, and, in particular, the oil press-cake, have been underutilized, often discarded as waste or used primarily for animal feed [12,13]. However, recent interest in the circular economy has highlighted the importance of fully exploiting agricultural by-products. Recent research indicates that the oil press-cake, for instance, is rich in protein, fiber, and bioactive compounds; thus, it holds considerable potential for applications in functional foods, nutraceuticals, and pharmaceutical formulations [13,14].

This study was conducted with the aim of characterizing and valorising one of the most important by-products of SI, the oil press-cake, which in this case was processed into powder. Far from being merely a residue of oil extraction, this by-product represents a promising source for sustainable applications. To this end, we analyzed its fatty acid and amino acid profiles, the presence of antinutritional factors, total phenolic content, and antioxidant capacity, in order to obtain a comprehensive overview of its chemical composition for food and/or feed applications. In addition, we provided novel evidence on the effect of the phenolic extract from *SI* oil press-cake powder (SICP) on cell viability, highlighting its potential for pharmaceutical and anti-tumor applications. To our knowledge, no previous work has combined compositional profiling with an evaluation of its selective bioactivity in human cell lines. Here, we provide the first integrated chemical–biological characterization of SIPC, a necessary step for future nutraceutical and technological developments. Hence, this research offers a comprehensive evaluation of Sacha inchi oil press-cake powder, underscoring its potential as a valuable resource for sustainable product development and as a nutritional and bioactive ingredient within a circular and resource-efficient framework.

## 2. Results and Discussion

### 2.1. Fatty Acid Profile

Determining the fatty acid composition allows us to assess the nutritional quality of the residual lipids in the oil press-cake. This information is crucial to evaluate its potential use as a functional ingredient in food or feed formulations, particularly regarding the presence of essential fatty acids and the balance between saturated and unsaturated fats. The fatty acid composition of SICP is summarized in Table 1, reporting the individual compounds, their concentrations (µg/g), and relative percentages. The analysis revealed that polyunsaturated fatty acids (PUFAs) represent the predominant class (74.94%), with α-linolenic acid (C18:3, ω-3, ALA) being the most abundant, accounting for over 40% of total fatty acids, followed by linoleic acid (C18:2, ω-6, LA) for over 30% (Table 1)

Monounsaturated fatty acids (MUFAs), mainly represented by oleic acid (C18:1), were detected at moderate levels, contributing to the oxidative stability of the matrix. Saturated fatty acids (SFAs), including palmitic acid (C16:0) and stearic acid (C18:0), were present in lower concentrations, which is nutritionally favorable.

Overall, α-linolenic acid, linoleic acid, and oleic acid were the most abundant fatty acids, corresponding to the PUFA and MUFA categories, respectively, for SICP. Other identified fatty acids were moreover present in minor amounts (Table 1). These results are consistent with previous studies on Sacha inchi oil, as reported by Alejandro Ruiz et al. [13] and Maurer et al. [15], who analyzed several authentic and commercial Sacha inchi oil samples and found comparable fatty acid compositions. Indeed, numerous studies have examined the fatty acid profile of Sacha inchi oil due to its recognized nutritional value; however, the oil press-cake fraction remains relatively unexplored, making its chemical characterization—particularly its fatty acid composition—of notable interest. The high content of α-linolenic acid (ALA, 42.52 ± 0.66%) and linoleic acid (LA, 32.43%) in the SICP (Sacha inchi oil press-cake powder) samples confirms the nutritional value of this by-product, as these are essential fatty acids that cannot be synthesized by the human body. This finding highlights Sacha inchi oil press-cake as a valuable plant-based source, especially of ω-3 fatty acids [16], with ALA levels surpassing those typically reported for fish oil—the primary source traditionally used for the extraction of ω-3 fatty acids—which range from 11.9% to 35.3% of total fatty acids [17]. This highlights the potential of Sacha inchi oil press-cake powder as an alternative source of essential ω-3 fatty acids for both nutritional and functional applications, including the development of new food and/or feed products.

### 2.2. Amino Acid Profile

The analysis of amino acids provides insight into the protein quality and potential biological value of SICP. This helps determine whether it can serve as a high-quality protein source for human consumption or animal nutrition.

Amino acid profiling indicated the presence of substantial quantities of both essential and non-essential amino acids. SICP contained 41.86% total protein, highlighting its high nutritional value. Among the most abundant non-essential amino acids were glycine (5.68%), aspartic acid (5.66%), glutamic acid (5.16%), and arginine (4.90%).

Essential amino acids were also present in appreciable quantities, including leucine (3.56%), valine (2.48%), and isoleucine (2.05%). Despite the overall richness in protein, methionine content was relatively low (0.81%) compared to other amino acids. As an essential amino acid, methionine’s lower concentration suggests the importance of dietary diversification to meet recommended daily intake levels. The total protein percentage was 41.86%, slightly lower than reported in previous studies [18]. All results are summarized in Table 2.

Considering amino acid content expressed as percentage (% *w*/*w*), the sample contains appreciable levels of essential amino acids, including leucine, valine, threonine, isoleucine, and phenylalanine, which are critical for multiple physiological functions and must be obtained through the diet. The total protein content is substantial, averaging 41.86%, and the amino acid profile is well-balanced, providing a wide spectrum of amino acids necessary for protein synthesis and diverse metabolic processes. Branched-chain amino acids (BCAAs) such as leucine, valine, and isoleucine are present in particularly high quantities, supporting muscle protein synthesis and energy production, which could be a potential application in functional food products, most commonly required by athletes and physically active individuals [19]. Non-essential amino acids, involved in neurotransmitter synthesis and energy metabolism, are also abundant, further enhancing the nutritional value of SI.

### 2.3. Antinutrient Factors

The antinutritional profile of SICP was evaluated to determine the presence and concentration of secondary metabolites with potential functional effects. The results are summarized in Table 3.

The analysis revealed that SICP contains measurable amounts of saponins, tannins, and alkaloids, which are classified as antinutritional compounds but are also recognized for their bioactive properties. The saponins were found in a concentration of 1050.1 ± 1.1 mg per 100 g SICP (DM), which indicates a significant occurrence. Tannins were detected at 6.2 ± 0.9 mg per 100 g (expressed as tannic acid), while alkaloids were present at 2.1 ± 0.5 mg per 100 g (expressed as berberine).

These findings suggest that, while SICP contains antinutritional metabolites that could limit direct consumption in large amounts, on the other hand these compounds could contribute to the functional properties of this matrix. Saponins, tannins, and alkaloids are known to exert bioactive effects, including antioxidant, antimicrobial, and potential anticancer activities, which may enhance the value of SICP beyond its nutritional profile.

On the other hand, the presence of antinutritional compounds may limit the direct application of SICP in food formulations [20,21]. But recent studies have demonstrated that hydrothermal treatments, including autoclaving (moist heat at 121 °C for 10–30 min) and hot-air sterilization, can significantly reduce various antinutrients. In particular, marked decreases were observed in alkaloids, nitrates, tannins, saponins, phytic acid, and trypsin inhibitors, with alkaloid reductions reaching up to 90% after 20 min of autoclaving. Beyond the reduction in antinutrients, these treatments also resulted in an increase in protein content (up to 70 g/100 g after 30 min of autoclaving), attributable to the concentration of nutrients and the formation of new protein structures following denaturation and aggregation processes [22]. However, these same compounds, tannins, saponins and alkaloids, have been reported to possess multiple pharmacological activities, including antimicrobial and selective cytotoxic effects [23]. The presence of these bioactive secondary metabolites in the current study is consistent with previous reports [20,21,22], suggesting that SICP could be valorized as a source of bioactive compounds for pharmaceutical or nutraceutical applications, in addition to its nutritional role.

### 2.4. Total Phenolic Content and Antioxidant Activity

Regarding TPC and AO, the highest values were obtained using the acetone/water/acetic acid (80/19/1 *v*/*v*/*v*) extraction mixture (Table 4). This observation is consistent with Chirinos et al. [24], who reported enhanced extraction yields using the same solvent system. The improved extraction efficiency is likely attributable to the content of condensed tannins in Sacha inchi oil press-cake powder, which are more effectively solubilized in acetone. Alternative solvents of varying polarities may also extract phenolic compounds, but often require elevated temperatures (>60 °C) to achieve comparable yields [25]. When acetone is employed as solvent, these temperature requirements can be lower, facilitating efficient extraction [25].

Since the present study aimed primarily to characterize the phenolic content in terms of TPC and AO rather than to optimize extraction yield, future investigations should focus on elucidating the interplay between solvent type and extraction temperature to maximize phenolic recovery from SICP.

Evaluation of phenolic compounds and antioxidant activity further supports the bioactive potential of Sacha inchi oil press-cake, as reported and highlighted in recent study [26]. In our case, the acetone/water/acetic acid extraction yielded the highest TPC and AO, attributable to condensed tannins and other phenolic compounds with known anti-inflammatory and antioxidant properties. In order to study the antioxidant capacity of the extracts obtained, three different tests were applied: DPPH, ABTS, and FRAP assays. This allows for a better investigation of the actual antioxidant capacity of the extracts, as reported by Thuanthong et al. [27] and Cardinali et al. [28]. The different assays applied for AO determination confirmed the superior radical scavenging capacity as the major potential in reducing ferric ions of the acetone-based extract, highlighting the importance of solvent choice and extraction parameters for optimizing phenolic yield and bioactivity. These results are in line with previous studies on SI leaves, where phenolic concentrations strongly depended on the extraction method [29].

### 2.5. Influence on Cellular Viability

**Figure 1:**
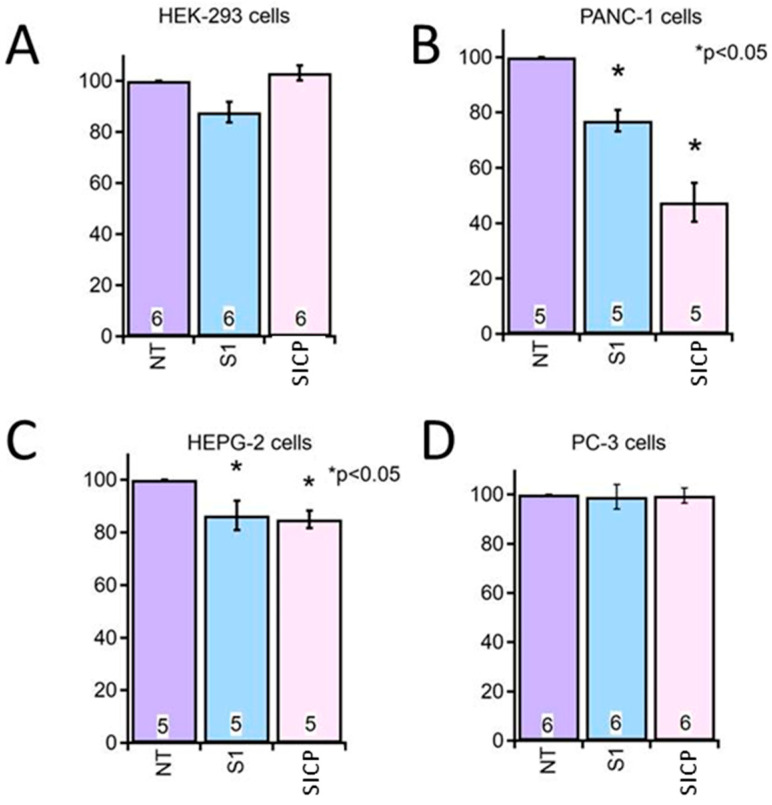
Effect of SICP Extract on Cell Viability in Different Cell Lines. Cell viability was assessed using the MTT assay following 72 h of incubation with SICP extract (SIPC, dilution 1:80) in different human cell lines: (**A**) HEK (human embryonic kidney, non-tumoral control), (**B**) Panc-1 (pancreatic ductal adenocarcinoma), (**C**) HepG2 (hepatocellular carcinoma), and (**D**) PC-3 (prostate adenocarcinoma). NT indicates untreated control cells; S1 indicates cells treated with the solvent mixture alone (acetone/water/acetic acid, 80/19/1 *v*/*v*/*v*), and SIPC indicates cells treated with SICP extracts. Results are expressed as percentage of cell viability relative to untreated controls (NT, 100% viability). Each bar represents the mean ± standard error of the mean (SEM), and the number inside each bar indicates the number of independent biological replicates performed. Statistical significance was evaluated by one-way ANOVA followed by Tukey’s post hoc test (* *p* < 0.05 vs. NT control).

For the cytotoxicity assays, the SICP extract selected was that obtained using the acetone/water/acetic acid (80/19/1 *v*/*v*/*v*) extraction mixture, which had yielded the highest TPC and AO in previous analyses. This extract did not induce any significant cytotoxicity in HEK cells, used as the non-tumoral control. Although the solvent alone (S1) exhibited some inherent cytotoxic effects, treatment with the SICP extract resulted in a significantly greater reduction in cell viability in Panc-1 (pancreatic adenocarcinoma) and HepG2 (hepatocellular carcinoma) cells compared to untreated controls (NT). Conversely, no significant changes in cell viability were observed in PC-3 (prostate adenocarcinoma) cells (Figure 1).

These findings suggest that the compounds contained in the SICP extract are bioactive and exert selective cytotoxic effects on specific human cancer cell lines, notably Panc-1 (pancreatic adenocarcinoma) and HepG2 (hepatocellular carcinoma), while having no significant impact on non-tumoral HEK cells or PC-3 prostate adenocarcinoma cells. This selective cytotoxicity, observed both between tumor and non-tumor cells and among different tumor types, indicates that the bioactive compounds may act through mechanisms influenced by the specific molecular and functional characteristics of each cell line, such as variations in redox balance, metabolic activity, or the expression of molecular targets involved in cell survival and proliferation. These observations align with previous reports highlighting the antiproliferative activity of specific chemical classes of compounds in SI, particularly flavonoids and tannins, which have been shown to modulate oxidative stress, interfere with inflammatory signaling pathways, and promote apoptosis in malignant cells [8,9,30,31]. The tumor-type-dependent response observed here underscores the need for further investigation to elucidate the molecular mechanisms underlying this selectivity and supports the rationale for continued exploration of SI-derived compounds as promising candidates in cancer research.

## 3. Materials and Methods

### 3.1. Raw Material

**Figure 2:**
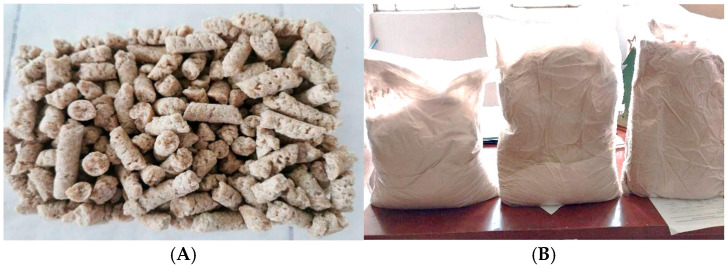
(**A**) Pellet-shaped *Sacha inchi* oil press-cake; (**B**) powdered *Sacha inchi* oil press-cake.

SI seeds were obtained from agricultural areas in the Province of Picota, San Martín region, Peru (6°54′21.7″ S 76°22′16.1″ W). The seeds were subjected to oil extraction by mechanical pressing at 28 °C, called “cold pressing”, a solvent-free process that ensures high-quality oil and produces the solid residue known as SI oil press-cake [32]. This residue was molded into pellets measuring 5 cm in length and 1 cm in diameter (Figure 2A), and subsequently oven-dried at 60 °C for 48 h. The dried pellets were ground in a mill (Model 4, Thomas Wiley brand grinder, NJ, USA) to obtain a powder with a particle size of 0.5 mm (Figure 1B). Finally, the powder (SICP) was vacuum-packed and stored at room temperature until analysis.

### 3.2. Fatty Acid Profile Determination

To characterize the fatty acid profile of SICP: a 5 g portion of powder was homogenized with n-hexane at room temperature for 30 min to extract the fat fraction. The mixture was filtered using a cellulose paper filter, and the solvent was removed with a rotary evaporator at 40 °C. The extracted fat was weighed, and 100 mg of it was dissolved in 8 mL of hexane. Then, 3 mL of 5% KOH in methanol was added. The mixture was shaken for 1 min to methylate the fatty acids, according to the method described by Annaratone et al. [33].

Subsequently, 0.9 mL of the upper phase of the solution was transferred into a vial, with the addition of 0.1 mL of a tricosanoic acid solution (3000 mg/kg in n-hexane) used as the internal standard. The fatty acids methyl esters (FAME) were analyzed using a Thermo Scientific Trace 1300 gas chromatograph (Thermo Scientific, Waltham, MA, USA) equipped with a Supelcowax (Bellefonte, PA, USA) fused silica capillary column (30 m × 0.25 mm × 0.25 µm film thickness). The system was coupled to a Thermo Scientific Trace ISQ mass spectrometer (Thermo Scientific, Waltham, MA, USA), featuring a single quadrupole analyzer. Helium was used as the carrier gas, and the injection was performed in split mode (split ratio = 1/20) at 280 °C.

The oven temperature program started at 60 °C, increasing to 280 °C at a rate of 20 °C/min after an initial hold of 3 min. The final temperature of 280 °C was maintained for 10 min, with a total run time of 24 min. Fatty acids were identified based on the comparison of their mass spectra with those in the NIST 2020 Mass Spectral Library (National Institute of Standards and Technology, Gaithersburg, MD, USA). Quantification was performed using the tricosanoic acid as the internal reference standard. Results are expressed as the mean of three replicates, calculated as µg/g of dried samples (DM = dry matter), and the relative percentages were reported. Then also the sum of saturated fatty acids (SFA), mono-unsaturated (MUFA) and poly-unsaturated (PUFA) fatty acids is presented.

### 3.3. Amino Acid Profile Determination

The total amino acid content was determined following the protocol outlined by Prandi et al. [34], with minor modifications. For general amino acid analysis, a 0.5 g sample of SICP was subjected to acid hydrolysis using 6 N hydrochloric acid (HCl) for 23 h at 110 °C (Sigma Aldrich, St. Louis, MO, USA). Nor-leucine (Sigma Aldrich, St. Louis, MO, USA) was added as an internal standard. For sulfur-containing amino acids such as methionine and cysteine, an additional oxidation step using performic acid was carried out prior to acid hydrolysis. Specifically, 50 mg of the sample was treated with freshly prepared performic acid (a mixture of 95% formic acid and hydrogen peroxide) and left at 0 °C for 16 h. Performic acid was then removed by adding hydrobromic acid, and samples were dried under nitrogen flow before undergoing acid hydrolysis.

A calibration curve was prepared using a 2.5 mM standard amino acid mixture (Thermo Scientific, Waltham, MA, USA) combined with 1:1 ratio of specific amino acids such as nor-leucine, hydroxyproline, cysteic acid, and methionine sulfone (Sigma Aldrich, St. Louis, MO, USA). This standard solution was diluted to create five concentration points (1.25 mM, 0.625 mM, 0.3125 mM, 0.156 mM, 0.078 mM) for calibration. Samples were derivatized using the AccQ-Fluor reagent kit (Waters, Milford, MA, USA), following the manufacturer’s instructions.

Amino acids were analyzed using a UPLC ACQUITY system coupled with an ACQUITY SQ ESI-MS system (Waters, Milford, MA, USA) equipped with a Peptide BEH C18 column (300 Å, 1.7 μm, 2.1 mm 170 × 150 mm) and a VanGuard™ (Agilent, Santa Clara, CA, USA) pre-column (300 Å, 1.7 μm, 2.1 mm × 5 mm). Data acquisition and processing were performed using MassLynx™ V4.0 software (Waters, Milford, MA, USA). Amino acid concentrations (%) were estimated from the calibration curves, using nor-leucine for normalization.

### 3.4. Antinutritional Factor Determination

Antinutritional factors are compounds naturally present in plants, such as tannins, saponins and alkaloids, and may also affect the bioavailability of potentially antioxidant compounds [35]. Their presence can reduce the nutritional value of plant-based foods by binding to essential minerals, inhibiting digestive enzymes, or affecting protein and carbohydrate metabolism [36]. Accurate determination of these compounds is essential for assessing the technological quality of SICP and optimizing subsequent processing methods to ensure its safety and nutritional adequacy in potential food or feed applications.

#### 3.4.1. Tannin Extraction and Quantification

Tannins were quantified according to the Folin–Ciocalteu method by Makkar H.P. [37], with slight modifications. A 0.4 g portion of SICP was mixed with 70 mL of distilled water and placed in an ultrasonic bath for 30 min. The mixture was then filtered using Whatman^®^ No. 2 filter paper, and the filtrate was brought to 100 mL with distilled water. A 1:10 dilution of the extract was prepared by mixing 50 μL of extract with 3950 μL of distilled water and 250 μL of Folin–Ciocalteu reagent. After 2 min, 750 μL of sodium carbonate (20% *v*/*v*) was added. The solution was protected from light and incubated for 1 h at room temperature. Absorbance was measured at 750 nm using a UV–Vis spectrophotometer (UV-1900i, Shimadzu, Tokyo, Japan). Tannin content was determined from a gallic acid calibration curve. Results were expressed as mg gallic acid equivalents per 100 g of dry matter (mg GAE/100 g DM).

#### 3.4.2. Saponin Extraction and Quantification

Saponin quantification was carried out according to the modified method by Guerra et al. [38]. For the preparation of the extract, 1 g of SICP was extracted with 10 mL of ethanol 80% (*v*/*v*) at a solid–liquid ratio of 1:10 (*w*/*v*) under constant agitation for 2 h at room temperature. The solution was then filtered, and the alcoholic extract was used for subsequent dilutions. This extractant was specific to the saponin assay and was not the same solvent mixture used for phenolic extraction. From this alcoholic extract, a 1:10 dilution was prepared using KH_2_PO_4_ buffer (pH 7.4). A series of eight dilutions (0.3–3.0 mL) was prepared and adjusted to a final volume of 6 mL with distilled water. Subsequently, 6 mL of sodium citrate solution (6%) and 1 mL of erythrocyte suspension were added to each tube. Tubes were incubated at 35 °C for 30 min, centrifuged at 2500 rpm for 5 min, and allowed to stand for 15 min. Absorbance was recorded at 540 nm using a UV–Vis spectrophotometer (UV-1900i, Shimadzu, Tokyo, Japan). Quantification was performed using a calibration curve of *Quillaja saponaria* saponins. Results were expressed as mg *Quillaja saponaria* equivalents per 100 g of dry matter (mg QSE/100 g DM).

#### 3.4.3. Alkaloids Extraction and Quantification

A spectrophotometric method based on reaction with bromocresol green (BCG) was used [39]. A 1 g portion of SICP was extracted with 20 mL of absolute methanol three times, using sonication for 20 min at 0 °C. The combined extracts were centrifuged at 4000 rpm for 15 min, filtered, and concentrated in a rotary evaporator. The residue was reconstituted in 20 mL of distilled water, basified to pH 12 with 10% ammonium hydroxide, and transferred to a separation funnel. Alkaloids were extracted into chloroform through repeated liquid–liquid extractions, monitored by thin-layer chromatography using Dragendorff’s reagent. The combined chloroform fractions were evaporated to dryness and reacted with BCG reagent. Absorbance was measured at 470 nm, and quantification was performed using a standard curve of berberine chloride (0–10 mg/L); and results were expressed as mg berberine equivalents per 100 g of dry matter (mg BE/100 g DM).

### 3.5. Total Phenolic Content and Antioxidant Activity Determination

For this investigation, two different extraction methods were used and compared with regard to the yield of phenolic compounds and antioxidant activity. The first extraction was performed following the method of Chiancone et al. [40]. An ethanol/water solution (80/20, *v*/*v*) with a solid–liquid ratio of 1:5 was used as the extracting mixture. The extraction was carried out with a shaker (HS 501 digital shaker, IKA-Werke GmbH & Co, Staufen im Breisgau, Germany) at 200 strokes/minute for 2 h at room temperature. For the second extraction, the procedure proposed by Chirinos et al. [24] was followed. A mixture of acetone/water/acetic acid (80/19/1, *v*/*v*/*v*) with a solid–liquid ratio of 1:10 was used to extract the target compounds. The extraction was performed for 60 min at 60 °C with a shaker incubator (SKI4, Shaking Incubator, ARGO LAB, Carpi, Italy). The extracts were centrifuged at 4000 rpm for 10 min at room temperature, and the supernatants were recovered to evaluate the TPC and the AO. Each extraction procedure was repeated twice. All results reported are expressed on dry matter (DM).

#### 3.5.1. Total Phenolic Content Determination

TPC was assessed using Folin–Ciocalteau’s phenol reagent, as described by Martelli et al. [41], with slight modifications. 250 µL of the extract was mixed with 1 mL of an aqueous solution of Folin–Ciocalteau’s phenol reagent (Sigma-Aldrich, St. Louis, MO, USA) at a ratio of 1:10 (*v*/*v*), followed by the addition of 2 mL of an aqueous solution of sodium carbonate (20%, *w*/*v*). The mixture was then kept in the dark for 30 min. The absorbance at 760 nm was measured using a spectrophotometer (JASCO V-530 spectrophotometer, Jasco, Easton, MD, USA). A calibration curve was generated by utilizing gallic acid as a reference compound across a concentration range of 10–100 mg/kg (5 data points) to determine the polyphenol content in the samples. Each sample extract was analyzed twice, and for enhanced precision, the instrument software was configured to perform three consecutive measurements on each sample. The same procedure was applied for other assays used to determine the antioxidant capacity of the extracts. The results for the total polyphenolic content were expressed as mg of gallic acid equivalents per gram of dry matter (mg GAE/g DM).

#### 3.5.2. Evaluation of Antioxidant Activity by DPPH, ABTS and FRAP Assays

AO of the extracts was assessed using the DPPH (2,2-Diphenyl-1-Picrylhydrazyl) radical scavenging assay, following the method described by Abram et al. [42] with minor adjustments. Specifically, 100 μL of either sample extract or standard solution was combined with 2.9 mL of a 0.05 mM ethanolic solution of DPPH (Sigma-Aldrich, St. Louis, MO, USA) and left in the dark for 30 min. Subsequently, the absorbance at 517 nm was measured using a JASCO V-530 spectrophotometer (Easton, MD, USA).

To determine the antioxidant capacity, 6-hydroxy-2,5,7,8-tetramethylchroman-2-carboxylic acid (Trolox) (Sigma-Aldrich, St. Louis, MO, USA) was employed as a reference compound, and five different standard solutions (0.1–1 mM) were prepared for constructing the calibration curve. Additionally, a blank containing 100 μL of the extraction solution was analyzed under the same conditions as the samples. The antioxidant capacity was calculated based on the percentage of radical inhibition (*I*%), using the formula:

$$I\%=100*\frac{\left(Ab_{B}-Ab_{S}\right)\;}{Ab_{B}}\;$$

where *Ab_B_* represents the absorbance of the blank and *Ab_S_* represents the absorbance of the sample/Trolox standard solution. The results were expressed as mg TEAC/mg (Trolox Equivalent Antioxidant Capacity). All analyses were performed in duplicate, with three consecutive measurements conducted on each sample.

Data obtained from the spectrophotometric tests were subjected to one-way Analysis of Variance, considering the factor “Extraction method” with a significance level of 0.05 (IBM SPSS Statistics 26.0 software, SPSS Inc., Chicago, IL, USA).

Similarly, ABTS [2,2-azinobis (3-ethylbenzothiazoline-6-sulfonic acid)] assay was performed following the indications given by Cardinali et al. [28]. The ABTS+ working solution was prepared by mixing ABTS (7 mM) and potassium persulfate (2.45 mM) in distilled water, and diluting this mixture with ethanol in order to obtain an ABTS solution with an absorbance of 0.7 measured at 734 nm. This solution was used to test the extract AO, using 20 μL of sample extract and 1980 μL of ABTS solution. After 30 min of reaction the absorbance values were measured at 734 nm with a JASCO V-530 spectrophotometer (Easton, MD, USA). AO was obtained against Trolox, as already described for DPPH assay.

Finally, Ferric-Ion-Reducing Power (FRAP) was tested, applying the same protocol reported by Cardinali et al. [28]. In particular, a FRAP working solution was created on the basis of a mixture of ferric chloride hexahydrate (20 mM) and 2,4,6-tripyridyl-s-triazine (TPTZ) (10 mM) aqueous solutions, by acidification with acetic acid, and heating at 37 °C. Then, this solution (2850 μL) was allowed to react with sample extracts (150 μL) for 30 min. The absorbance was measured at 593 nm (JASCO V-530 spectrophotometer, Easton, MD, USA) and compared with that of Trolox, using the same calibration curve described for DPPH and ABTS procedures.

### 3.6. Effect of Phenolic Extract on Cell Viability

To investigate the potential bioactivity of SICP extracts, bioactive compounds were extracted using an acetone/water/acetic acid solvent mixture (80/19/1, *v*/*v*/*v*), which enabled a higher recovery of TPC and enhanced AO in the DPPH assay. After extraction, as previously described, the samples were centrifuged at 4000 rpm for 10 min at room temperature. The resulting supernatant was collected and diluted at a 1:80 ratio for use in cell-based assays.

Panc-1 (human pancreatic ductal adenocarcinoma), HepG2 (human hepatocellular carcinoma), Calu-3 (human lung adenocarcinoma), PC-3 (human prostate adenocarcinoma), and HEK (human embryonic kidney) cell lines were maintained in appropriate growth media following ATCC recommendations. Specifically, Panc-1 and HEK cells were cultured in Dulbecco’s Modified Eagle Medium (DMEM), HepG2 and Calu-3 cells in Minimum Essential Medium (MEM) supplemented with 1% non-essential amino acids, and PC-3 cells in DMEM/F-12 (1:1) medium. All media were supplemented with 10% fetal bovine serum (FBS), 1% penicillin-streptomycin and 2 mM L-glutamine. Cells were incubated at 37 °C in a humidified atmosphere containing 5% CO_2_.

Cell viability was evaluated using the MTT assay, a well-established method to assess cellular metabolic activity [43]. This colorimetric assay measures the enzymatic reduction in the tetrazolium salt MTT (3-[4,5-dimethylthiazol-2-yl]-2,5-diphenyltetrazolium bromide) to insoluble purple formazan crystals by mitochondrial oxidoreductase enzymes, reflecting viable cell function. Cells were incubated with the diluted SICP extract for 72 h. A solvent control (acetone/water/acetic acid, 80/19/1 *v*/*v*/*v*), identical to the extraction solution but without the extract, was included to distinguish specific cytotoxic effects of the phytochemicals. After incubation, MTT reagent was added, and following standard incubation, formazan absorbance was measured spectrophotometrically at 570 nm on a multiplate reader (TriStar2 S LB 942 Multimode Reader, Berthold Technologies, Bad Wildbad, Germany) to determine relative cell viability.

The data are expressed as mean ± standard error of the mean (SEM), calculated from a minimum of five independent biological replicates. Statistical significance of differences among multiple treatment groups was determined using one-way analysis of variance (ANOVA), followed by Tukey’s post hoc test for pairwise comparisons. Statistical analyses were performed with IgorPro 9 software (Lake Oswego, OR, USA). Differences were considered statistically significant at a *p*-value < 0.05.

## 4. Conclusions

Taken together, these results suggest that SI oil press-cake, often considered a by-product, is a rich source of essential nutrients and bioactive compounds with multifunctional potential. Its ω-3 fatty acids, balanced amino acid profile, phenolic content, antioxidant capacity and selective cytotoxic activity argue for its valorisation as a component of functional food and/or feed, nutraceuticals and pharmaceutical applications, especially when formulated in powder form. Importantly, this work provides the first integrated chemical–biological characterization of SIPC, linking its compositional profile with selective effects on human cell viability. By combining detailed nutrient and antinutrient analysis with multi-line cytotoxicity assays, this study fills a critical gap in the current literature and offers new evidence supporting the biological relevance of SIPC-derived extracts. This integrated perspective strengthens the rationale for the technological and nutraceutical exploitation of this underutilized resource.

Future studies should focus on the isolation and characterization of individual phenolic constituents with antioxidant activity, optimization of extraction techniques for large-scale application and evaluation of bioavailability and safety to fully exploit the industrial potential of Sacha inchi oil press-cake powder.

## Figures and Tables

**Table 1:** Fatty acids profile (%) of *Sacha inchi* oil press-cake powder.

Fatty Acid		*m*/*z*	% ± SD
Palmitic acid	C16:0	270	5.14 ± 0.37
Palmitoleic acid	C16:1	268	0.09 ± 0.06
Margaric acid	C17:0	284	0.15 ± 0.01
Margaroleic acid	C17:1	282	0.06 ± 0.01
Stearic acid	C18:0	298	4.57 ± 0.12
Oleic acid	C18:1	296	14.21 ± 1.23
Linoleic acid	C18:2	294	32.43 ± 0.89
Linolenic acid	C18:3	292	42.52 ± 0.66
Arachidic acid	C20:0	326	0.16 ± 0.01
Unsaturated arachidic acid	C20:1	324	0.69 ± 0.00
**SFA**			10.01 ± 0.26
**MUFA**			15.05 ± 1.30
**PUFA**			74.94 ± 1.55

Results are expressed as the mean ± standard deviation of three replicates, and are reported as relative percentages (%). Then the sum of saturated fatty acids (SFA), mono-unsaturated (MUFA) and poly-unsaturated (PUFA) fatty acids is presented.

**Table 2:** Amino acids profile (%) of *Sacha Inchi* oil press-cake powder.

Amino Acid	Relative Percentage (% *w*/*w*) ± SD
Glycine	5.68 ± 0.54
Alanine	1.81 ± 0.07
Serine	3.51 ± 0.18
Proline	2.15 ± 0.06
Valine	2.48 ± 0.08
Threonine	2.50 ± 0.16
Isoleucine	2.05 ± 0.09
Leucine	3.56 ± 0.14
Aspartic Acid *	5.66 ± 0.76
Lysine	1.54 ± 0.16
Glutamic Acid **	5.16 ± 0.69
Histidine	1.40 ± 0.17
Phenylalanine	1.48 ± 0.26
Arginine	4.90 ± 0.43
Tyrosine	2.71 ± 0.50
Methionine	0.81 ± 0.10
Cysteine	1.68 ± 0.15
**Total Proteins** %	41.86 ± 1.64

* The sum of aspartic acid and asparagine; ** The sum of glutamic acid and glutamine.

**Table 3:** Antinutritional metabolites of *Sacha inchi* oil press-cake powder.

Chemical Component	mg/100 g DM
Tannins	6.2 ± 0.9 *
Saponins	1050.1 ± 1.1 **
Alkaloids	2.1 ± 0.5 ***

Results are expressed as means ± SD (standard deviation). DM = dry matter of SICP. * Tannins expressed as mg gallic acid equivalents per 100 g DM (mg GAE/100 g DM). ** Saponins expressed as mg *Quillaja saponaria* equivalents per 100 g DM (mg QSE/100 g DM). *** Alkaloids expressed as mg berberine equivalents per 100 g DM (mg BE/100 g DM).

**Table 4:** Total phenolic content (TPC) and antioxidant activity (AO) measured by DPPH, ABTS and FRAP.

Extraction Method	TPC (mg GAE/g)	DPPH (mg TEAC/g)	ABTS (mg TEAC/g)	FRAP (mg TEAC/g)
Ethanol/water (80/20 *v*/*v*)	0.18 ± 0.01 ^b^	0.19 ± 0.01 ^b^	0.39 ± 0.16	0.84 ± 0.04
Acetone/water/acetic acid (80/19/1 *v*/*v*/*v*)	0.47 ± 0.02 ^a^	0.35 ± 0.01 ^a^	1.28 ± 0.33	1.13 ± 0.05

Results are expressed as means ± SD: TPC values are reported as gallic acid equivalents (GAE), while antioxidant capacity is reported as Trolox anti-oxidant equivalents (TEAC). Means within a column with different letters are significantly different (*p* < 0.01).

## Data Availability

The original contributions presented in this study are included in the article. Further inquiries can be directed to the corresponding author.

## Abbreviations

The following abbreviations are used in this manuscript:

ABTS	2,2′-azinobis(3-ethylbenzothiazoline)-6-sulfonic acid
AO	Antioxidant activity
BCAAs	Branched-chain amino acids
Calu-3	Human lung adenocarcinoma
DM	Dry matter or Sample of SICP
DMEM	Dulbecco’s Modified Eagle Medium
DPPH	2,2-diphenyl-1-picrylhydrazyl hydrate
FAME	Fatty acids methyl esters FBS
HepG2	Human hepatocellular carcinoma
HEK	Human embryonic kidney
*m/z*	Mass-to-charge ratio
MEM	Minimum Essential Medium
MTT	3-(4,5-dimetiltiazol-2-il)-2,5-difeniltetrazolio bromuro assay
MUFA	Mono-unsaturated fatty acids
Panc-1	Human pancreatic ductal adenocarcinoma
PC-3	Human prostate adenocarcinoma
PUFA	Poly-unsaturated fatty acids
SD	Standard deviation
SI	Sacha inchi
SICP	Sacha inchi oil press-cake powder
SFA	Saturated fatty acids
TEAC	Trolox Equivalent Antioxidant Capacity
TPC	Total phenolic content
Trolox	6-hydroxy-2,5,7,8-tetramethylchroman-2-carboxylic acid
